# P07-04 A systematic review of key interventional elements in international exercise referral schemes

**DOI:** 10.1093/eurpub/ckac095.104

**Published:** 2022-08-29

**Authors:** Eriselda Mino, Inga Naber, Sarah Klamroth, Anja Weissenfels, Wolfgang Geidl, Peter Gelius, Karim Abu-Omar, Klaus Pfeifer

**Affiliations:** Department of Sport Science and Sport, Friedrich-Alexander University Erlangen-Nürnberg (FAU), Erlangen, Germany

**Keywords:** exercise referral scheme, exercise prescription, physical activity on prescription scheme, physical activity promotion, primary health care

## Abstract

**Background:**

With the first initiatives dating back to the 1990s, the past two decades have seen a rapid increase in the use of exercise referral schemes (ERS) worldwide. Despite the accumulating evidence on their effectiveness, there are currently no international guidelines available to inform the design of such interventions. The key elements and processes employed vary both within and between countries. This systematic review aims to address this frequently overlooked topic by identifying elements that are predominant in international ERS.

**Methods:**

Scientific databases (PubMed, Scopus) and grey literature sources were systematically searched. In order to collect the information relevant for understanding and visualizing all ERS models, a broad spectrum of document types was considered eligible for inclusion, i.e. randomized controlled or pragmatic trials, cohort studies, case-control studies, case series, case reports, qualitative studies, economic evaluations, mixed designs, policy documents, and official governmental reports. We extracted data on scheme components, contents, and main actors involved in scheme delivery. Cross-functional flowcharts were employed to facilitate comparison between different ERS designs: Firstly, the collected data were visualized in flowcharts indicating the pathway a patient follows from beginning to end of an individual ERS. Secondly, elements that appeared more frequently across all included ERS were identified.

**Results:**

Preliminary results identified 18 models of ERS that were eligible for data analysis, including Green Prescription (New Zealand), Hreyfiseðill (Iceland), National Exercise Referral Scheme (Wales). Program designs ranged from short advice by a primary healthcare professional to physical activity prescription and/or further referral to affiliated health professionals. The prevailing actors involved in scheme delivery were physicians, nurses, physiotherapists, training experts, physical activity providers, and coordinators. Seven predominant elements emerged from the comparison between ERS designs: assessment, counselling, individualized physical activity recommendations, written prescription, behavior change techniques, support person, and follow-up.

**Conclusions:**

To the best of our knowledge, this is the first study that takes a closer look at the design characteristics of ERS across the world. Our preliminary results indicate that there are seven key elements. The contribution of these elements on the effectiveness of ERS needs to be explored in future research.

